# The Relationship between Serum Vitamin D Level and Attention Deficit Hyperactivity Disorder

**Published:** 2015

**Authors:** Mohammad Reza SHARIF, Mahla MADANI, Fatemeh TABATABAEI, Zakieh TABATABAEE

**Affiliations:** 1 Department of Pediatrics, Kashan University of Medical Sciences, Kashan, Iran; 2 Student of Research Committee, Kashan University of Medical Sciences, Kashan, Iran; 3 Isfahan Endocrine & Metabolism Research Center, Isfahan University of Medical Sciences, Isfahan, Iran; 4 Department of Clinical Psychology, Kashan University of Medical Sciences, Kashan, Iran

**Keywords:** Vitamin D, Children, Psychiatric diseases, ADHD

## Abstract

**Objective:**

Attention deficit hyperactivity disorder (ADHD) is one of the most prevalent mental health disorders. In recent years, the impacts of various micronutrients on ADHD have been studied. However, vitamin D has received much less attention. This study was aimed at evaluating the association and level of serum vitamin D in children with ADHD.

**Materials & Methods:**

This case-control study was carried out, in 2012, on 6 to 12 yr-old children. Thirty-seven were children with ADHD in the cases group and another 37 healthy children were in the control group. Venous blood sample was drawn from each child to measure the serum level of vitamin D. Other variables were compared as well.

**Results:**

The mean serum vitamin D level of children with ADHD (19.11±10.10 ng/ml) was significantly lower than that of the control group (28.67±13.76 ng/ml) (P<0.001).

**Conclusion:**

Deficiency of vitamin D has been proved in various psychiatric diseases. This study evidenced a significantly low level of serum vitamin D in children with ADHD. This suggests the need for regularly monitoring of serum vitamin D levels and treatment of patients with vitamin D deficiencies.

## Introduction

Attention deficit hyperactivity disorder (ADHD) is one of the most prevalent mental health disorders that affect about 5.3–7.1 percent of children and adolescents (1). Attention deficiency, hyperactivity and impulsivity are three main symptoms that help diagnose the disorder before the age of twelve years (1, 2). Besides, other accompanying secondary symptoms such as aggression, social incompetence, conflict with peers and anti-social behavior other clinically important symptoms (2, 3). So far, drug therapy is the main treatment method. However, there are limitations in drug interventions. For instance, 30 percent of ADHD children do not respond to the drug treatment (4, 5). More effective treatment and strategies are needed to control the disease (6, 7). In recent years, the role of environment (8-10) and more specifically the role of nutrition in the prevention and treatment of the symptoms of the disease have been attracting the attention of researchers (11-13). Diet therapy is a simple and inexpensive method that can be readily accepted by the parents and adopted by the children. Nutrition therapy, especially the role of supplements and vitamins is very pronounced (11). The role of micronutrients such as iron (14-16), zinc (17, 18) and omega three (19) on the prevention and control of the symptoms have been extensively studied. However, the role of vitamin D has attracted less attention. This condition exists despite the fact that vitamin D deficiency is associated with psychiatric diseases such as autism, schizophrenia, and depression (20-22). This vitamin is not only involved in bone metabolism and serum calcium regulation but also has significant effect on many body organs (23). Thus, it has been recommended that the level of this vitamin should be checked in pregnant women and their babies (20, 24). Vitamin D deficiency during the fetal period is accounted for some psychological disorders after birth (21). In addition, it has been suggested that vitamin D insufficiency during the fetal and post-natal period has unfavorable effects on the development of the brain structure and functions (20). This study was designed to examine serum vitamin D level of children diagnosed to have ADHD.

## Materials & Methods

This case-control study was carried out in 2012. Healthy children and children diagnosed to have ADHD in the age range of 6 to 12 year-old were eligible to enter the study. Overall, following the completion of consent form, 74 children voluntarily participated in the study. The case group (n=37) included children who had ADHD medical records at a pediatric psychiatric clinic in Isfahan and the control group (n=37) among non ADHD children selected from those who were referred to health centers for checking their weight and height. Their demographic data were collected at the time of referral. A psychiatrist has ruled out the ADHD and diagnosis was made based on facts from a face-to-face interview with the child and his parents and using the Statistical Manual of Mental Disorders Diagnostic (DSM-IV) criteria (25). After confirming the diagnosis of ADHD, a child would enter the study protocol. A child who suffered from liver, kidney or any endocrine disease or who was on vitamin D supplement was excluded from the study. Besides, a child diagnosed to have mental retardation, autism or seizures was excluded. Parents were explained about the safety and purpose of the study and about confidentiality of every child’s data. This study was approved by Ethic Committee of Kashan University of Medical Science. After obtaining written informed consent from each participant’s parent, three milliliter (ml) of venous blood was drawn from each child in both the cases and control groups. Standard DIA source kit and ELISA methods were used to measure the level of 25-hydroxi vitamin D. The serum vitamin D level was classified as severely low (< 10 nanograms (ng) per ml), low (10 to 30 ng/ml), normal (30 to 100 ng/ ml) or toxic level (> 100 ng/ml) based on the analysis of the blood (26, 27). Data were analyzed using the statistical packages for social sciences (SPSS) version 16.0 (Chicago, IL, USA). Independent t-test and chi square tests were performed and any association was considered significant at an alpha level of ≤0.05. The parents were informed about if their children found to have below or above normal serum vitamin D levels.

## Results

There was no statistically significant difference between the cases and control groups based on age and gender of the study participants. However, there was a statistically significant difference (P < 0.001) in serum vitamin D levels between the cases and the control groups (Table 1). The serum vitamin D level of 21.6% children in the cases (ADHD) group was normal. The remaining 78.4% children had serum vitamin D level below normal. In the control group, 48.6% of the children had normal serum vitamin D level. None of the children in both groups showed toxic level of vitamin D. Analysis has shown a statistically significant difference (P=0.04) in serum vitamin D levels between the ADHD and the control groups (Table 2). Furthermore, there was no a statistically significant difference (P=0.3) in serum vitamin D level between boys and girls (21.25±11.52 vs. 21.25±11.52) in the ADHD group of children. Similarly, there was no a statistically significant difference (P=0.9) in the serum vitamin D level of boys and girls (28.71±13.22 vs. 28.63±14.61) in the control group. Besides, there was no a statistically significant age difference (P=0.5) between the children with serum vitamin D level less than 30 ng/ ml (9.14±2.33) and children with normal serum vitamin D level (9.50±2.17).

**Table 1:** Comparison of the relationship of sex, age and Vitamin D levels (ng/ml) with ADHD and Control groups

**Variables** **Condition**	**Gender**		**Age (yr)**		**Vitamin D level (ng/ml)**	
	**Boy**	**Girl**	**Mean**	**SD**	**Mean**	**SD**
ADHD	23 (62%)	14 (38.7%)	9.13	2.37	19.11	10.10
Control	18 (48.6%)	19 (51.4%)	9.40	2.19	28.67	13.76
Total	41 (55.4%)	33 (44.6%)	9.27	2.27	23.89	12.92
P	0.2		0.6		0.001	

**Table 2.** Comparison of serum vitamin D levels of ADHD and control groups

**Variables** **Condition**	**Serum vitamin D levels (ng/ml)**		
	**Severe deficient** **n (%)**	**Deficient** **n (%)**	**Normal** **n (%)**
ADHD	11 (29.7)	18 (48.6)	8 (26.1)
Control	6 (16.2)	13 (35.1)	18 (48.6)
Total	17 (23)	31 (41.9)	26 (35.1)
P	0.04		

## Discussion

The results of the present study indicated that the proportion of children with vitamin D deficiency in ADHD group was significantly larger than that of the normal children. In addition, the mean value of serum vitamin D level in the cases group (19.11±10.10) was significantly lower than in the control (healthy) group (28.67±13.76). This finding is consistent with the report of a similar study conducted in Turkey among 7 to 18 years old children where a significant difference (P < 0.05) in mean serum vitamin D level between cases (20.9±19.4 ng/ml) and control groups (34.9±15.4 ng/ ml) was demonstrated (28). Another study on 1331 cases of ADHD and the same number of control groups healthy individuals under the age of 18 found out that the mean range serum vitamin D level of ADHD children (16.6±7.8 ng/ml) was lower than in the control group (23.5±9.9 ng/ml). In addition, 8.15% of the ADHDs had normal vitamin levels in their serum (29). These results are also similar to what was found in our study as previously stated. In an interventional study on 80 patients with ADHD above the age of 16 in New Zealand, reported 27% prevalence rate of vitamin D deficiency. Using vitamin D supplement for eight weeks was found to be effective in alleviating the signs of the disease. However, adding other micronutrients such as zinc, vitamin B12, iron and folate was not found effective (30). In contrast, another study in England reported no significant association between some behavioral problems including ADHD and vitamin D level (31). The incidence of ADHD is much lower in areas with sunny weather and sunlight can have a protective effect against the disease (32). Phototherapy and sunray have been used as a treatment (33, 34). A hypothesis in this regard is that sunrays increase the level of vitamin D level (32). Several studies have been conducted to identify the role of nutrition on the prevalence of the disease, however, many of them have emphasized on the role of breast feeding. These studies assume that the nutrient contents of breast milk such as vitamins, minerals, etc. may act as protective agents. However, there is a need for further investigations (35-38). Various studies have examined the effects of micronutrients such as iron, zinc and omega 3 on ADHD, but there is limited evidence suggesting the association between vitamin D deficiency and ADHD. Thus, further studies seem necessary (14-19, 39). Although little research has implicated the relationship between ADHD and vitamin D, extensive studies have investigated the role of this vitamin in other psychiatric/ neurologic disorders (40, 41) including Alzheimer’s disease (42) depression (43, 44), schizophrenia (45) and autism (46). Since vitamin D is a neurosteroid, lack of this vitamin results in many psychological disorders (20). In addition, vitamin D plays a protective role in brain health, that is, it increases the expression of transpeptidasec-glutamyl. This enzyme enhances the formation of glutathione, which is the most important brain antioxidant factor (47). Lack of this vitamin during the fetal life and childhood, in the very early days of life affect the nerve differentiation, axon synapses, brain structure, and function (20). In the present research, although a considerably high percentage of children in the ADHD had vitamin D deficiency, healthy children in the control was also found to have suffered from vitamin D deficiency. Vitamin D deficiency is a worldwide epidemic. Despite the abundance of sunlight, the prevalence of vitamin D deficiency in countries located around the Persian Gulf region is high. Findings indicated that 70% of very young girls in Iran and 80% in Saudi Arabia suffered from vitamin D deficiency (48). Exposure to sunlight alone is not sufficient to alleviate vitamin D deficiency despite sun light plays a role in maintaining vitamin D level in blood (49).

**In conclusion**, the low levels of serum vitamin D among the ADHD children suggest the need for regularly monitoring of serum vitamin D levels and treatment of patients with vitamin D deficiencies. In addition, life style and diet should be modified and directed towards eliminating the nutritional deficiencies in the society.

## Notes

**Acknowledgements** The authors would like to appreciate kind assistance and financial support provided by the Vice Chancellor for Research at Kashan University of Medical Sciences. We thank the parents of our patients for their cooperation and permission to publish this study.
